# Spider (Arachnida, Araneae) fauna of the lowland part of the Balkhash-Alakol Basin (SE Kazakhstan): an integrated assessment

**DOI:** 10.3897/BDJ.14.e181501

**Published:** 2026-02-20

**Authors:** Anna Nekhaeva, Leonid Kim, Anel Ishayeva, Anatoly Ozernoy, Artëm Sozontov

**Affiliations:** 1 Institute of Zoology RK, Almaty, Kazakhstan Institute of Zoology RK Almaty Kazakhstan; 2 A.N. Severtsov Institute of Ecology and Evolution, Russian Academy of Sciences, Moscow, Russia A.N. Severtsov Institute of Ecology and Evolution, Russian Academy of Sciences Moscow Russia; 3 Institute of Plant and Animal Ecology UB RAS, Ekaterinburg, Russia Institute of Plant and Animal Ecology UB RAS Ekaterinburg Russia

**Keywords:** arid ecosystems, iNaturalist, GBIF, biodiversity, open data, Central Asia, occurrence

## Abstract

**Background:**

Despite more than a century of research (since 1896 to the present), a comprehensive summary of the spider fauna of the lowland part of the Balkhash-Alakol Basin is still lacking. The scattered and fragmentary nature of the available data hampers a thorough assessment of the species diversity and zoogeographical features of this territory.

**New information:**

For the first time, based on original material, published scientific sources and records from open-access resources (GBIF, iNaturalist), a comprehensive assessment of the diversity of the spider fauna in the lowland and foothill arid parts of the Balkhash-Alakol Basin has been carried out. To date, this is the only and most complete list of spiders in the region.

Original records are presented, including rare and little-known species for the region, with refined data on their ranges. The quality of open-access data was evaluated and its main shortcomings were identified. It is shown that, with the involvement of experienced naturalists who maintain contact with specialists, the proportion of reliable records can be significantly higher. The results broaden our knowledge of spider biodiversity in the region and may serve as a basis for future faunistic and zoogeographical studies.

## Introduction

Deserts are unique ecosystems inhabited by organisms highly adapted to extreme environmental conditions. These species often form isolated populations and display distinctive survival strategies (Gilyarov 1970, Cloudsley-Thompson 1983, Cloudsley-Thompson 2001). Despite their relatively low species richness, deserts are characterised by a high level of endemism, making species loss in these environments more significant for biodiversity compared to species-rich biomes (McNeely 2003, Maestre et al. 2021). However, deserts are often mistakenly perceived as barren or degraded landscapes requiring reclamation, which diminishes interest in studying and conserving their biodiversity (Zhang et al. 2023).

The spider fauna of the deserts of the Balkhash Region (the lowland part of south-eastern Kazakhstan) remains poorly studied to this day. The first information on spiders from the region was published by Schmidt (1896) in his work on the fauna of the Semirechye Province of the Russian Empire, where he recorded six spider species from the City of Vernyi (the former name of Almaty) and adjacent areas. Later, based on long-term faunistic studies, a paper on the spider fauna of Kazakhstan was published, which included records of 133 species found in Almaty and its surroundings (Spassky and Shnitnikov 1937). In the following decades, studies of the arachnofauna in this region, as in most parts of Kazakhstan, were mainly taxonomic in nature and relied on incidental or short-term collections (see Suppl. material 1). This resulted in fragmented knowledge scattered across numerous publications, which still hampers a comprehensive assessment of the spider diversity of the region. Even the presence of a checklist of spider species of Russia and adjacent countries (Mikhailov 2024a) does not fully address this issue, as each species included in the checklist is assigned only to major physiographical areas. This spatial assignment does not reflect the actual distribution of species within specific regions and natural complexes, thereby significantly limiting analyses of regional diversity and comparative biogeographic studies.

At the present stage, new material on the spider fauna of the region has been collected by the staff of the Institute of Zoology of the Republic of Kazakhstan. Part of the material was also deposited in the collection of the Institute by A. Ozernoy. Amongst the collected specimens are noteworthy records that supplement or refine existing knowledge of the species composition of spider communities in the deserts of the Balkhash Region. In addition, this region is currently the best represented in open-access sources: of more than 4,000 spider occurrence records in Kazakhstan, nearly 2,400 come from the Balkhash-Alakol Basin (GBIF.org 2025). This makes the region a key area for analysing the representativeness of open data and assessing the state of biodiversity in the country’s arid territories.

The aim of this study is to provide a comprehensive assessment of the species diversity of spiders in the lowland deserts of the Balkhash Region, based on original material, published scientific sources and records from open-access resources (GBIF, iNaturalist). We also compare these sources to evaluate their completeness, consistency and suitability for describing the spider fauna of the region.

## General description

### Additional information

The article presents two datasets on spider (Araneae) records from the lowland and foothill arid parts of the Balkhash-Alakol Basin. The territories of Almaty City and Talgar settlement (excluding their mountainous areas above 950 m a.s.l.) are also included, as they contain a significant number of records. The first dataset compiles scattered literature data on spider records from the study area (Nekhaeva et al. 2025a), while the second contains original data recently collected by the staff of the Institute of Zoology of the Republic of Kazakhstan (Nekhaeva et al. 2025b). Both datasets include qualitative and quantitative information. In cases where literature sources did not provide the number of collected specimens, only qualitative data are presented.

The literature dataset (Nekhaeva et al. 2025a) includes 1,507 occurrences, 1,250 of which belong to the plain part of the studied region and will be further considered. The remaining 257 occurrences come from the mountainous part (950 m a.s.l. and above) and are, therefore, excluded from the analysis. To date, this is the most comprehensive summary of literature data on the spider fauna of the Balkhash-Alakol Basin, covering the period from 1896 to 2023.

The IZRK collection dataset (Nekhaeva et al. 2025b) includes 543 occurrences, collected and identified mainly during 2023–2025, with additional material from 2019–2022 also included. Almost all identifications were carried out primarily to the species level.

To make the most complete list of spiders in the region, we also use open-access occurrence records from the GBIF portal, including the dataset "iNaturalist research-grade observations". We treat this dataset separately from all other GBIF data, since its records are primarily based on amateur observations and identifications, whereas the other datasets originate from academic institutions.

Taken together, these materials provide a comprehensive and representative foundation for analysing the composition of the spider fauna of the arid part of the Balkhash-Alakol Basin.

## Project description

### Title

Spider (Arachnida, Araneae) fauna of the lowland part of the Balkhash-Alakol Basin (SE Kazakhstan)

### Personnel

Anna Nekhaeva, Leonid Kim, Anel Ishayeva, Aidyn Yeszhanov, Anatoly Ozernoy, Artëm Sozontov.

### Funding

Committee of Science of the Ministry of Science and Higher Education of the Republic of Kazakhstan, Grant No. BR21882199 «Cadastre of wild animals of arid territories of the Balkhash-Alakol Basin with an assessment of threats for their conservation and sustainable use».

## Sampling methods

### Sampling description

**Field studies**

Field studies were conducted in 2019–2025 across various desert types (sandy, gravelly, clay and rocky) and plant communities (wormwood, saltwort, semi-shrub, shrub etc.) at elevations up to 950 m a.s.l. Spiders were collected mainly by hand (including at night), as well as with pitfall traps (plastic cups, 200 ml volume, 65 mm opening diameter, filled to one-third with a 7% vinegar solution or a 4% formalin solution). The total number of adult spiders collected during this period was 805 specimens, amongst which 183 species were identified.

**Literature mining and digitisation of published sources**

The search and selection of literature containing information on spiders of the studied region were carried out mainly using the Bibliography on Spiders of Russia and the Republics of the post-Soviet Republics (Mikhailov 2024b), through its digital mirror and search engine “Arachnolibrary” (Sozontov and Mikhailov 2024a, Sozontov and Mikhailov 2024b). In addition, literature searches were performed using Google Scholar and the World Spider Catalog (World Spider Catalog 2025). All arachnological papers with the words “Kazakhstan” or “Казахстан” in their titles were reviewed. Publications that did not mention specific spider species were excluded. If information on the number of collected specimens was absent, only the presence of a species was recorded in the summary table. Literature data were extracted using the web platform Faunistics 2.0, exporting human-readable into machine-readable tables, following the DarwinCore standard (Sozontov 2024).

In total, we processed 74 references published between 1896 and 2023, 66 of them containing occurrences from the studied region. All literature data accumulated in these 66 references (Suppl. material 1) contain 1,259 records of 263 species. Most of these are taxonomic papers, including descriptions of new taxa, but some faunistic summaries are also present.

**Analysis of open data sources**

Open data were obtained through GBIF.org applying taxa (order = Araneae) and spatial (country = KZ) filters (GBIF.org 2025). Next, it was specified manually with the following filters: identification level (not less than "GENUS"), event date (<2025-04-18), elevation (< 950 m a.s.l.) and the focus area (lowland part of the Balkhash-Alakol Basin, spatial filter) with further separation into the two parts: the dataset "iNaturalist research-grade observations" (amateur data) and all other datasets (academician data) with 2,361 and 27 occurrences, respectively. Code and spatial data files are available on the GitHub repository.

### Quality control

All collected spiders are preserved in 90–95% alcohol and stored in the arachnid collection of the Institute of Zoology of the Kazakhstan Republic (IZRK). Almost all specimens were identified to species. Species identification was also carried out on juvenile specimens in cases where there were no doubts (for example, if juveniles were collected together with adults). Species identification was performed by A. Nekhaeva and L. Kim using numerous taxonomic publications. Some definitions that raised questions were checked by D.V. Logunov (St. Petersburg, Russia) and G.N. Azarkina (Novosibirsk, Russia). Taxonomy nomenclature complies with the World Spider Catalog (World Spider Catalog 2025).

Coordinates were not provided for all literature records. Therefore, georeferencing was carried out independently using Soviet topographic maps. In all cases, the accuracy of the coordinates in metres was specified, corresponding to the degree of confidence in the georeferencing.

To verify amateur data, we checked the validity of each identification. For this purpose, photographs of every iNaturalist record were reviewed by arachnology specialists (A. Nekhaeva and L. Kim) and the identifications were assigned to one of five categories: 1) reliable beyond doubt (the photo shows clearly visible copulatory organs or another distinct trait allowing species-level identification); 2) highly likely correct (external features are sufficient for identification); 3) questionable (blurry photos); 4) insufficient for identification (e.g. poor angle, bad lighting); 5) incorrect identification. For verification, we also used additional photographs of the species uploaded to iNaturalist by the observer, information on whether the record had been reviewed by other arachnologists and, in some cases, examination of preserved material. As a result, records assigned to categories 1–2 were considered valid, those in category 3 as doubtful and those in categories 4–5 as invalid.

All taxonomy was corrected according to the World Spider Catalog (World Spider Catalog 2025).

### Step description

1. Collection of field material and its identification;
2. Search and selection of literature;
3. Literature data digitisation;
4. Georeferencing of collection sites in cases where this information was not provided in the text;
5. Downloading data for the study region from the GBIF portal, verification of the obtained records and compilation of a verified species list;
6. Comparison of all obtained species lists.

## Geographic coverage

### Description

The surveyed territory lies within the administrative borders of the Almaty and Jetisu Regions and partly includes the Karaganda and Jambyl Regions of Kazakhstan (see the interactive map). It covers lowland and foothill areas (up to 950 m a.s.l.), stretching from south to north — from the Zailiysky Alatau Mts and the middle reaches of the Ili River valley to Northern Balkhash — and from west to east, from the Chu-Ili Mts to the eastern shore of Lake Alakol (excluding the Dzhungarian Alatau Mts). The territories of Almaty City and Talgar settlement within the specified altitudinal range were also included in the study region, as they have the longest history of research and the highest number of records. At present, these are amongst the most populated areas with the greatest number of occurrence records in open-access databases.

### Coordinates

43.04 and 46.85 Latitude; 73.77 and 81.36 Longitude.

## Taxonomic coverage

### Description

Spiders

### Taxa included

**Table:** 

Rank	Scientific Name	
kingdom	Animalia	
phylum	Arthropoda	
class	Arachnida	
order	Araneae	

## Temporal coverage

### Notes

The literature dataset covers the period from 1896 to 2023.

The IZRK collection dataset covers the period from 2 September 2019 and 14 May 2025.

The dataset “iNaturalist research-grade observations” covers the period from 01-01-2000 to 17-04-2025, which is an top limit manually applied (see below).

The GBIF datasets covers the period from 13-08-1929 to 24-04-2016.

## Usage licence

### Usage licence

Open Data Commons Attribution License

### IP rights notes

other

## Data resources

### Data package title

Spider (Arachnida, Araneae) fauna of the lowland part of the Balkhash-Alakol Basin (SE Kazakhstan)

### Resource link

https://doi.org/10.15468/7qrc2k; https://doi.org/10.15468/5wwewh

### Alternative identifiers

https://www.gbif.org/dataset/92cc4b3a-97f2-4aa3-b260-883ba061c1a9; https://www.gbif.org/dataset/791d91fe-ce27-4044-8a3d-a2a8b203f979

### Number of data sets

2

### Data set 1.

#### Data set name

Spider (Arachnida, Araneae) fauna of the lowland part of the Balkhash-Alakol Basin (SE Kazakhstan). Part 1: Literature data

#### Data format

Darwin Core

#### Download URL

http://gbif.ru:8080/ipt/resource?r=almaty_literature

#### Description

The spider (Araneae) records from the lowland and foothill arid parts of the Balkhash-Alakol Basin, based on the literature sources (Nekhaeva et al. 2025a).

The territories of Almaty City and Talgar settlement (excluding their mountainous areas above 950 m a.s.l.) are also included, as they contain a significant number of records. The dataset compiles scattered literature data (66 articles about fauna and taxonomy) from the study area and includes qualitative and quantitative information. In cases where literature sources did not provide the number of collected specimens, only qualitative data are presented.

The dataset includes 1,507 occurrences, 1,250 of which belong to the plain part of the studied region and will be further considered. The remaining 257 occurrences come from the mountainous part (950 m a.s.l. and above) and are, therefore, excluded from the analysis. To date, this is the most comprehensive summary of literature data on the spider fauna of the Balkhash-Alakol Basin, covering the period from 1896 to 2023.

**Data set 1.** 

Column label	Column description
datasetName	The name identifying the dataset from which the record was derived. A constant ("Spider (Arachnida, Araneae) fauna of the lowland part of the Balkhash-Alakol Basin (SE Kazakhstan). Part 1: Literature data").
type	The nature or genre of the resource. A constant ("Text").
basisOfRecord	The specific nature of the data record. A constant ("MaterialCitation").
modified	Date on which the resource was changed. A constant ("2025-12-05").
language	A language of the resource. A constant ("en|ru").
license	A legal document giving official permission to do something with the resource. A constant ("CC-BY").
bibliographicCitation	A bibliographic reference for the resource. A variable.
references	A related resource that is referenced, cited or otherwise pointed to by the described resource. A variable.
rightsHolder	A person or organisation owning or managing rights over the resource. A constant ("Institute of Zoology of the National Academy of Sciences, Republic of Kazakhstan (IZRK)").
institutionID	An identifier for the institution having custody of the object(s) or information referred to in the record. A constant ("https://scientific-collections.gbif.org/institution/7e82dc97-c81e-4361-845f-4338170452b2").
institutionCode	The name (or acronym) in use by the institution having custody of the object(s) or information referred to in the record. A constant ("https://zool.kz/").
dynamicProperties	A list of additional measurements, facts, characteristics or assertions about the record. A variable. Notifies if the record belongs to plain ("plain" value) or mountain ("mountain" value) of the studied region.
occurrenceID	An identifier for the dwc:Occurrence. A variable.
occurrenceStatus	A statement about the presence or absence of a dwc:Taxon at a dcterms:Location. A constant ("present").
individualCount	The number of individuals present at the time of the dwc:Occurrence. A variable.
sex	The sex of the biological individual(s) represented in the dwc:Occurrence. A variable.
lifeStage	The age class or life stage of the dwc:Organism(s) at the time the dwc:Occurrence was recorded. A variable.
recordedBy	A list of names of people, groups or organisations responsible for recording the original dwc:Occurrence. A variable.
occurrenceRemarks	Comments or notes about the dwc:Occurrence. A variable.
associatedReferences	A list of identifiers of literature associated with the dwc:Occurrence. A variable.
verbatimEventDate	The verbatim original representation of the date and time information for a dwc:Event. A variable.
eventDate	The date-time or interval during which a dwc:Event occurred. A variable.
habitat	A category or description of the habitat in which the dwc:Event occurred. A variable.
samplingProtocol	The names of, references to or descriptions of the methods or protocols used during a dwc:Event. A variable.
eventRemarks	Comments or notes about the dwc:Event. A variable.
continent	The name of the continent in which the dcterms:Location occurs. A constant ("Asia").
countryCode	The standard code for the country in which the dcterms:Location occurs. A constant ("KZ").
country	The name of the country or major administrative unit in which the dcterms:Location occurs. A constant ("Kazakhstan").
verbatimLocality	The original textual description of the place. A variable.
verbatimCoordinates	The verbatim original spatial coordinates of the dcterms:Location. A variable.
decimalLatitude	The geographic latitude of the geographic centre of a dcterms:Location. A variable.
decimalLongitude	The geographic longitude of the geographic centre of a dcterms:Location. A variable.
geodeticDatum	The ellipsoid, geodetic datum or spatial reference system (SRS), upon which the geographic coordinates are based. A constant ("WGS84").
coordinateUncertaintyInMeters	The horizontal distance (in metres) from the given dwc:decimalLatitude and dwc:decimalLongitude describing the smallest circle containing the whole of the dcterms:Location. A variable.
georeferencedBy	A list of names of people, groups or organisations who determined the georeference for the dcterms:Location. A variable.
georeferencedDate	The date on which the dcterms:Location was georeferenced. A variable.
minimumElevationInMeters	The lower limit of the range of elevation, in metres. A variable.
maximumElevationInMeters	The upper limit of the range of elevation, in metres. A variable.
kingdom	The full scientific name of the kingdom in which the dwc:Taxon is classified. A constant ("Animalia").
phylum	The full scientific name of the phylum or division in which the dwc:Taxon is classified. A constant ("Arthropoda").
class	The full scientific name of the class in which the dwc:Taxon is classified. A constant ("Arachnida").
order	The full scientific name of the order in which the dwc:Taxon is classified. A constant ("Aranea").
family	The full scientific name of the family in which the dwc:Taxon is classified. A variable.
genus	The full scientific name of the genus in which the dwc:Taxon is classified. A variable.
scientificName	The full scientific name, with authorship and date information if known. A variable.
verbatimIdentification	A string representing the taxonomic identification as it appeared in the original record. A variable.
taxonRank	The taxonomic rank of the most specific name in the dwc:scientificName. A variable.
taxonRemarks	Comments or notes about the taxon or name. A variable.
typeStatus	A list of nomenclatural types applied to the subject. A variable.
identifiedBy	A list of names of people, groups or organisations who assigned the dwc:Taxon to the subject. A variable.
dateIdentified	The date on which the subject was determined as representing the dwc:Taxon. A variable.
catalogNumber	An identifier for the record within the dataset or collection. A variable.

### Data set 2.

#### Data set name

Spider (Arachnida, Araneae) fauna of the lowland part of the Balkhash-Alakol Basin (SE Kazakhstan). Part 2: IZRK collection data

#### Data format

Darwin Core

#### Download URL

http://gbif.ru:8080/ipt/recourse?r=almaty_collection

#### Description

The spider (Araneae) records from the lowland and foothill arid parts of the Balkhash-Alakol Basin, based on spiders collection of Institute of Zoology of the Republic of Kazakhstan (Nekhaeva et al. 2025b).

The territories of Almaty City and Talgar settlement (excluding their mountainous areas above 950 m a.s.l.) are also included, as they contain a significant number of records. The dataset contains original data recently collected by the staff of the Institute of Zoology of the Republic of Kazakhstan.

The IZRK collection dataset includes 543 occurrences, collected and identified mainly during 2023–2025, with additional material from 2019–2022 also included. Almost all identifications were carried out primarily to the species level.

**Data set 2.** 

Column label	Column description
type	The nature or genre of the resource. A constant ("PhysicalObject").
modified	Date on which the resource was changed. A constant ("2025-10-27").
language	A language of the resource. A constant ("en").
license	A legal document giving official permission to do something with the resource. A constant ("CC-BY").
rightsHolder	A person or organisation owning or managing rights over the resource. A constant ("Institute of Zoology of the National Academy of Sciences, Republic of Kazakhstan (IZRK)").
institutionID	An identifier for the institution having custody of the object(s) or information referred to in the record. A constant ("https://scientific-collections.gbif.org/institution/7e82dc97-c81e-4361-845f-4338170452b2").
institutionCode	The name (or acronym) in use by the institution having custody of the object(s) or information referred to in the record. A constant ("https://zool.kz/").
datasetName	The name identifying the dataset from which the record was derived. A constant ("Spider (Arachnida, Araneae) fauna of the lowland part of the Balkhash-Alakol Basin (SE Kazakhstan). Part 2: IZRK collection data").
basisOfRecord	The specific nature of the data record. A constant ("PreservedSpecimen").
occurrenceID	An identifier for the dwc:Occurrence. A variable.
occurrenceStatus	A statement about the presence or absence of a dwc:Taxon at a dcterms:Location. A constant ("present").
disposition	The current state of a dwc:MaterialEntity with respect to a collection. A constant ("in collection").
recordNumber	An identifier given to the dwc:Occurrence at the time it was recorded. A variable.
fieldNumber	An identifier given to the dwc:Event in the field. A variable.
individualCount	The number of individuals present at the time of the dwc:Occurrence. A variable.
recordedBy	A list of names of people, groups or organisations responsible for recording the original dwc:Occurrence. A variable.
sex	The sex of the biological individual(s) represented in the dwc:Occurrence. A variable.
lifeStage	The age class or life stage of the dwc:Organism(s) at the time the dwc:Occurrence was recorded. A variable.
eventDate	The date-time or interval during which a dwc:Event occurred. A variable.
verbatimEventDate	The verbatim original representation of the date and time information for a dwc:Event. A variable.
year	The four-digit year in which the dwc:Event occurred, according to the Common Era Calendar. A variable.
month	The integer month in which the dwc:Event occurred. A variable.
day	The integer day of the month on which the dwc:Event occurred. A variable.
georeferencedDate	The date on which the dcterms:Location was georeferenced. A variable.
georeferencedBy	A list of names of people, groups or organisations who determined the georeference for the dcterms:Location. A variable.
identificationRemarks	Comments or notes about the dwc:Identification. A variable.
habitat	A category or description of the habitat in which the dwc:Event occurred. A variable.
samplingProtocol	The names of, references to or descriptions of the methods or protocols used during a dwc:Event. A variable.
continent	The name of the continent in which the dcterms:Location occurs. A constant ("Asia").
country	The name of the country or major administrative unit in which the dcterms:Location occurs. A constant ("Kazakhstan").
countryCode	The standard code for the country in which the dcterms:Location occurs. A constant ("KZ").
stateProvince	The name of the next smaller administrative region than country in which the dcterms:Location occurs. A variable.
locality	The specific description of the place. A variable.
locationRemarks	Comments or notes about the dcterms:Location. A variable.
minimumElevationInMeters	The lower limit of the range of elevation, in metres. A variable.
maximumElevationInMeters	The upper limit of the range of elevation, in metres. A variable.
decimalLatitude	The geographic latitude of the geographic centre of a dcterms:Location. A variable.
decimalLongitude	The geographic longitude of the geographic centre of a dcterms:Location. A variable.
geodeticDatum	The ellipsoid, geodetic datum or spatial reference system (SRS), upon which the geographic coordinates are based. A constant ("WGS84").
coordinateUncertaintyInMeters	The horizontal distance (in metres) from the given dwc:decimalLatitude and dwc:decimalLongitude describing the smallest circle containing the whole of the dcterms:Location. A variable.
identifiedBy	A list of names of people, groups or organisations who assigned the dwc:Taxon to the subject. A variable.
dateIdentified	The date on which the subject was determined as representing the dwc:Taxon. A variable.
scientificName	The full scientific name, with authorship and date information if known. A variable.
kingdom	The full scientific name of the kingdom in which the dwc:Taxon is classified. A constant ("Animalia").
phylum	The full scientific name of the phylum or division in which the dwc:Taxon is classified. A constant ("Arthropoda").
class	The full scientific name of the class in which the dwc:Taxon is classified. A constant ("Arachnida").
order	The full scientific name of the order in which the dwc:Taxon is classified. A constant ("Araneae").
family	The full scientific name of the family in which the dwc:Taxon is classified. A variable.
genus	The full scientific name of the genus in which the dwc:Taxon is classified. A variable.
specificEpithet	The name of the first or species epithet of the dwc:scientificName. A variable.
scientificNameAuthorship	The authorship information for the dwc:scientificName formatted according to the conventions of the applicable dwc:nomenclaturalCode. A variable.
taxonRank	The taxonomic rank of the most specific name in the dwc:scientificName. A variable.
county	The full, unabbreviated name of the next smaller administrative region than stateProvince in which the dcterms:Location occurs. A variable.
taxonRemarks	Comments or notes about the taxon or name. A variable.
verbatimCoordinates	The verbatim original spatial coordinates of the dcterms:Location. A variable.
collectionCode	The name, acronym, coden or initialism identifying the collection or dataset from which the record was derived. A constant ("IZRK_Ara").

## Additional information

### Мaterial and methods

Digital photographs were taken at the Joint Usage Center “Instrumental Methods in Ecology”, A.N. Severtsov Institute of Ecology and Evolution, Moscow (IEE RAS), using a Keyence microscope and at the Laboratory of Arachnology and Other Invertebrates, Institute of Zoology, Almaty (IZRK), using a Leica S APO stereomicroscope with a Canon EOS 90D DSLR camera; image stacking was performed using Helicon Focus 8.3 software.

Photographs of live spiders and their copulatory organs were taken using a Canon EOS 750D camera, a Canon EF 100 mm f/2.8L Macro IS USM lens and a Yongnuo YN-24EX Macro TTL flash.

### Results

**Literature data**

We digitised 66 taxonomic and faunistic publications published between 1896 and 2023. As doubtful records in the final checklist of spider species of the region (Table 1), *Alopecosa latifasciata* (Kroneberg, 1875) and *Lycosa tarantula* (Linnaeus, 1758), reported by Spassky and Shnitnikov (1937), were excluded. The taxonomy of the former species remains very complicated and requires re-examination of Spassky’s material and/or the study of newly-collected material (see Logunov (2023)). *L. tarantula* inhabits the Mediterranean Region and is currently reliably known only from Italy; all other records of the species are highly doubtful (D. Logunov, personal communication). In addition, *Xysticus cristatus* (Clerck, 1757) was excluded from the list, since this temperate species with a Euro-Siberian range was, with high probability, confused with *X. pseudocristatus*, which inhabits a variety of arid and forest habitats in Central Asia and was described later (Azarkina and Logunov 2001). Conversely, *Eresus tristis* was retained in the list, despite the fact that Marusik and Azarkina (2020) suggested that the record of this species from south-eastern Kazakhstan (see Spassky and Shnitnikov (1937)) may represent a misidentification and actually belong to an undescribed related species.

Thus, the literature sources provide data on 263 species from 115 genera and 25 families.

**GBIF data**

There are critically few well-georeferenced records on GBIF — only 27 entries, originating from sequencing/barcoding projects (boldsystems.org – 1 record; www.ebi.ac.uk – 3 records) or from digitised literature data (Plazi – 10 records), based on three sources (Zonstein and Marusik 2016, Fomichev 2022, Logunov and Ponomarev 2020, Fomichev 2022,), as well as several specimens (9) from the Naturmuseum Senckenberg. This highlights the critical under-representation of the spider fauna of Kazakhstan on global biodiversity data portals and, consequently, the urgent need to fill this gap. Taking into account records without coordinates, only a few additional occurrences can be attributed to the area of interest, based on their descriptions, but they do not change the overall pattern. Thus, the data presented in this work increase by nearly two orders of magnitude the volume of open-access GBIF records on spiders of Kazakhstan originating from academic institutions.

Thus, the academician data from GBIF.org provide 15 species from eight genera and five families.

**iNaturalist data**

From the “iNaturalist Research-grade Observations” dataset, we obtained information on 2,361 spider records. Amongst them, 12% (288 records) were identified by us as doubtful, lacking sufficient data for identification or misidentified.

The following 17 species were not included in the final checklist, based on iNaturalist data (Table 1): *Aculepeira armida*, *Alopecosa cursor*, *Archaeodictyna consecuta*, *Cheiracanthium punctorium*, *Dictyna arundinacea*, *Drassodes lapidosus*, *Larinioides ixobolus*, *L. patagiatus*, *Micaria formicaria*, *Microlinyphia pusilla*, *Metleucauge dentipalpis*, *Pardosa zonsteini*, *Phlegra obscurimagna*, *Talavera aperta*, *Tetragnatha montana*, *Trochosa ruricola* and *Theridion melanurum*, as their identification was classified as questionable (Category 3 in the “Quality Control” subsection).

Another 16 species, not recorded in any other sources, were also excluded from the final checklist (Table 1) (*Callilepis nocturna* (Linnaeus, 1758), *Eresus kollari* Rossi, 1846, *Euryopis flavomaculata* (C.L.Koch, 1836), *Heriaeus horridus* Tystshenko, 1965, *H. oblongus* Simon, 1918, *Heterotheridion nigrovariegatum* (Simon, 1873), *Hypsosinga kazachstanica* Ponomarev, 2007, *Larinia phthisica* (L.Koch, 1871), *Neoscona spasskyi* (Brignoli, 1983), *Pardosa falcata* Schenkel, 1963, *Platnickina tincta* (Walckenaer, 1802), *Pseudomogrus bucharaensis* (Logunov & Marusik, 2003), *Runcinia tarabayevi* Marusik & Logunov, 1990, *Sibianor aurocinctus* (Ohlert, 1865), *Thanatus fabricii* (Audouin, 1826) and*Thanatus mongolicus* (Schenkel, 1936)), since their identification to species level, based on photographs, is doubtful and cannot currently be confirmed otherwise. *Fedotovia mongolica* Marusik, 1993 was also excluded and replaced with *F. uzbekistanica*, as the former was described from a female. The published photographs of palps, combined with the absence of females in the collections, do not allow us to reliably distinguish the collected specimen from *F. uzbekistanica*. The latter occurs in south-western and southern Kazakhstan and is also known from Uzbekistan, Tajikistan and Afghanistan (Fomichev and Marusik 2015); thus, its record in the Balkhash Region is expected. In addition, *Alopecosa fedotovi* (Charitonov, 1946) and *Alopecosa hui* Chen, Song & Kim, 2001 were replaced in the list (Table 1) with *Alopecosa* sp., as they presumably represent a species new to science. For the same reason, *Evippa beschkentica* Andreeva, 1976 was replaced with E. aff. caucasica.

With some reservations, since photographs do not allow for unambiguous conclusions, but with preliminary confirmations from specialists, 10 species were included in the final checklist (Table 1): Aelurillus cf. nenilini, Bassaniodes cf. turlan, Bolephthyphantes cf. indexoides, Enoplognatha cf. latimana, Entelecara cf. erythropus, Neottiura cf. bimaculata, Ozyptila cf. tuberosa, Philodromus cf. longipalpis, Theridion cf. mystaceum and Xysticus cf. kuzgi. All of them were recorded only on iNaturalist and not in literature sources or original material.

Thus, the iNaturalist platform includes 194 species from 102 genera and 25 families.

**Original data**

The checklist based on original data included *Dysdera* sp., *Pritha* sp., *Agyneta* sp., *Steatoda* sp. and *Xysticus* sp., as they are distinct from other recorded species. Thus, the original collections yielded 183 species from 103 genera and 26 families.

**New findings**

**Figure 1.**
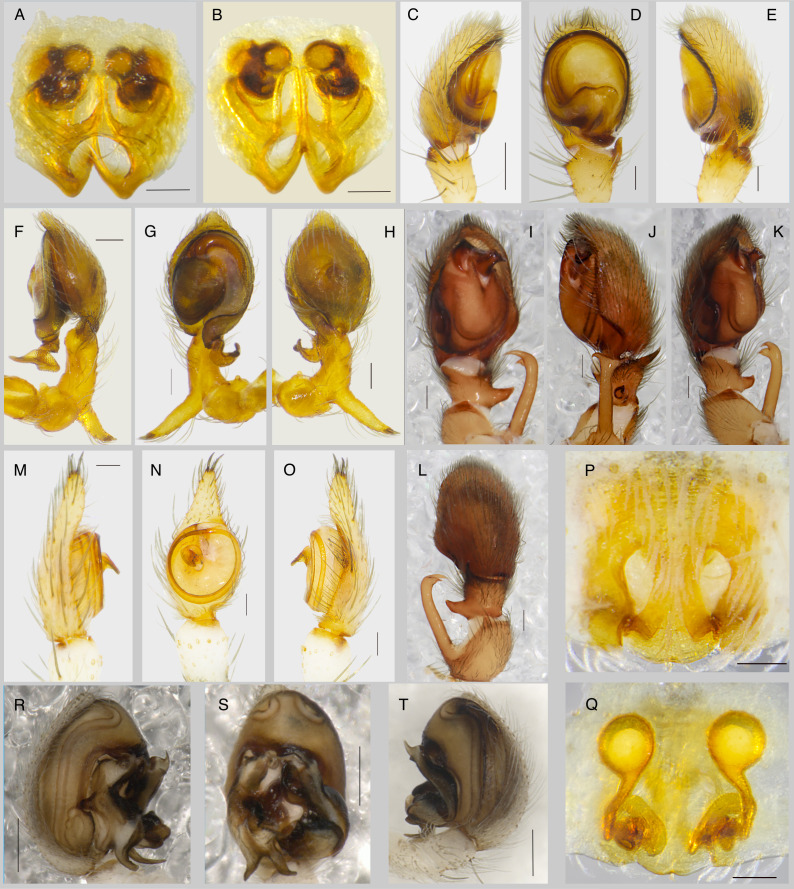
Copulatory organs of *Porrhoclubiona laudata* (A-E), *Tolkienus ottoi* (F-H), *Coreodrassus recepsahini* (I-L), Hersiliola cf. korbi (M-O), *Evippa beschkentica*, (P, Q), *Turanobius leptonychus* (R-T): **A, P** – epigyne, ventral; **B, Q** – epigyne, dorsal; **C, K, M, R** – palp, prolateral; **D, G, I, N, S** – palp, ventral; **E, F, J, O, T** – palp, retrolateral; **H, L** – palp, dorsal. Scale bars: A-B, D-H, M-T – 0.2 mm; C, I-L 0.4 mm.

Amongst the newly-collected material and open-source records, 24 species are new for Kazakhstan (18 and 11 species, respectively, with five species shared between both lists) (Table 1). Amongst them, 14 species seem to be the most interesting:

*Porrhoclubiona laudata* (O. Pickard-Cambridge, 1885) is distributed in south-western Xinjiang (China) and adjacent north-eastern Jammu and Kashmir (India) (Marusik and Omelko 2018). The female epigyne resembles that of *P. leucaspis* (Simon, 1932), while the vulva differs in the size of the receptacle (Fig. 1A-E).

*Tolkienus ottoi* (Marusik & Koponen, 2017) (Fig. 1F-H) was originally described from Azerbaijan (Marusik and Koponen 2017) and later reported from other parts of the Caucasus (Dagestan, Russia; Georgia) and Iran (Caspian Sea coast) (Zamani et al. 2021, World Spider Catalog 2025, Zamani et al. 2021). Our record from Almaty represents the easternmost occurrence and extends the known range of the species by nearly 30° in this direction.

*Coreodrassus recepsahini* Coşar, Danışman & Marusik, 2024 (Fig. 1I-L) was recently described and, until now, has been known only from Turkiye (Anatolia) (Coşar et al. 2024). The record from the Balkhash Region extends the known range of the species 30° eastwards. Considering such a significant distributional disjunction, it is reasonable to assume that *C. recepsahini* also inhabits several intermediate regions and future findings may be expected from the Caucasus, Iran, Turkmenistan and Uzbekistan.

Hersiliola cf. korbi Fomichev, 2025 (Fig. 1M-O) was recently described from Kyrgyzstan, based on a single male. Our record from Almaty represents one of the northernmost occurrences of the family Hersilidae (Fomichev 2025).

**Figure 2.**
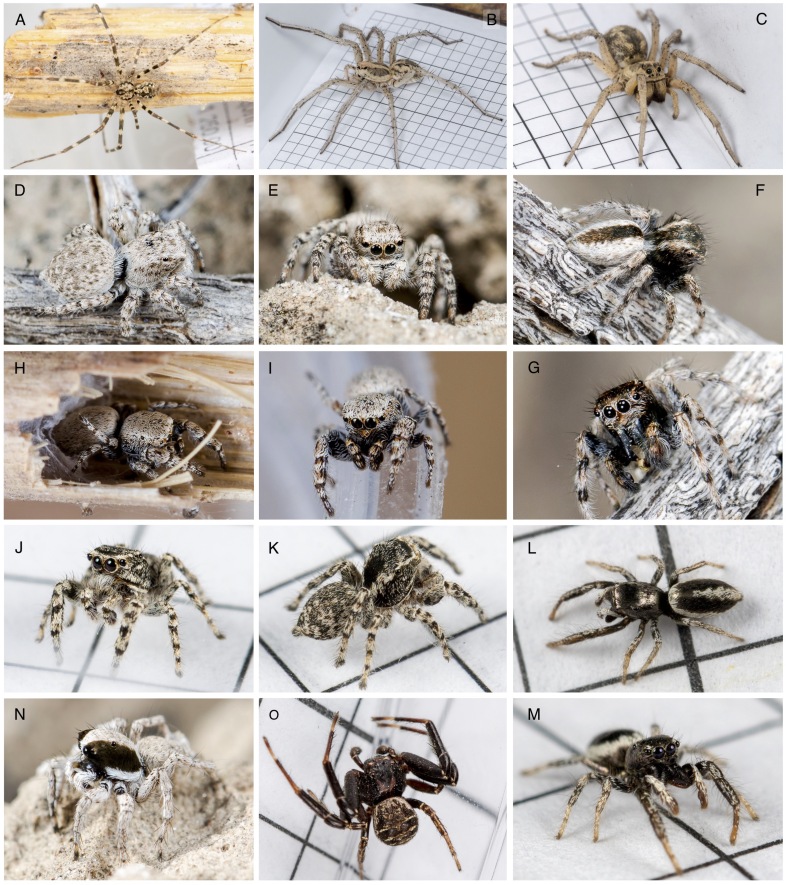
Habitus of living individuals: *Hersiliola xinjiangensis* male (A), Karakumosa cf. xinjiang male (B) & female (C), *Aelurillus andreevae* female (D, E) & male (N), *Mogrus valerii* male (F, G), *Pseudomogrus bactrianus* male (H, I), *Pseudomogrus mirabilis* male (J, K), *Salticus karakumensis* male (L, M), *Xysticus pseudoluctuosus* male (O).

**Figure 3.**
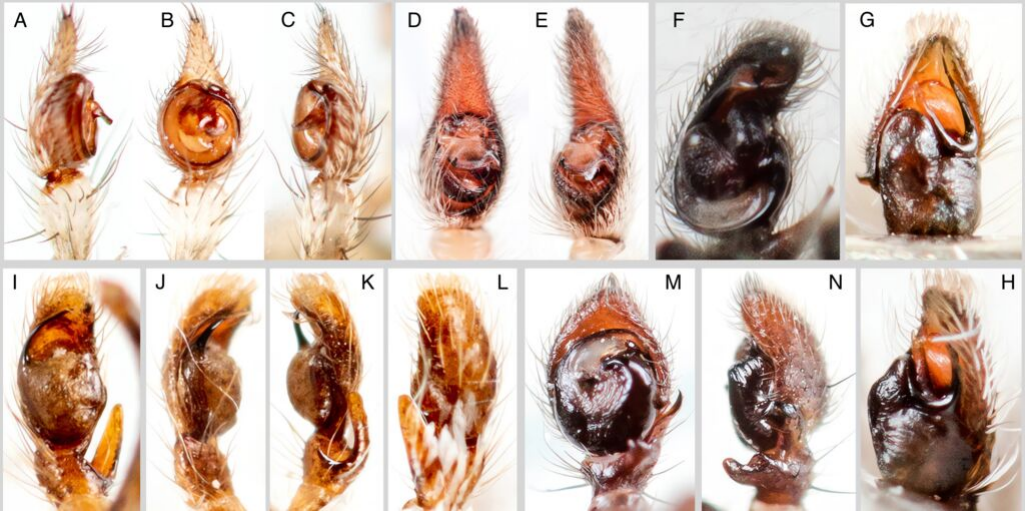
Copulatory organs of living individuals of *Hersiliola xinjiangensis* (A-C), Karakumosa cf. xinjiang (D, E), *Mogrus valerii* (F), *Pseudomogrus mirabilis* (G-H), *Salticus karakumensis* (I-L), *Xysticus pseudoluctuosus* (M, N). **A, J** – palp, prolateral; **B, D, F, G, I, M** – palp, ventral; **C, E, H, K, N** – palp, retrolateral; **L** – palp dorsal.

*Hersiliola xinjiangensis* (Liang & Wang, 1989) (Fig. 2A) is known from China (Xinjiang, Urumchi) (Liang and Wang 1989). According to Marusik and Fet (2009), the type material is presumably lost. Only a single original illustration of this species exists and all subsequent publications mentioning *H. xinjiangensis* have reproduced the figures from the original description (see World Spider Catalog (2025)). We provide the first photograph of the male palp of this species since its description (Fig. 3A-C). The records of *H. xinjiangensis* from Almaty also represent some of the northernmost occurrences of the family Hersilidae (Fomichev 2025).

*Evippa beschkentica* Andreeva, 1976 (Fig. 1P and Q) is known from Tajikistan (World Spider Catalog 2025). The record from the Balkhash Region is the first since the species was originally described.

Karakumosa cf. xinjiang Wang, Yang & Zhang, 2023 (Fig. 2B, C, Fig. 3D and E) was recently described from China (Xinjiang Uygur Autonomous Region, Huocheng County, Liushiliu Huolongdong) (Wang et al. 2023). Our record was made relatively close to the type locality of this species.

*Turanobius leptonychus* Zamani, Marusik & Fomichev, 2024 (Fig. 1R-T) was recently described from south-western Tajikistan (World Spider Catalog 2025). Our record from the vicinity of Almaty is the first since the original description and extends the known range of the species eastwards.

*Aelurillus andreevae* Nenilin, 1984 (Fig. 2D and E) was designated, but not described by Nenilin (1984), based on material from Turkmenistan and Tajikistan. The records from the Balkhash Region and the middle reaches of the Ili River represent the first for Kazakhstan. A re-description of this species is currently being prepared by G. Azarkina, who identified it in iNaturalist.

*Mogrus valerii* Kononenko, 1981 (Fig. 2F, G and Fig. 3F) is known from Turkmenistan and Uzbekistan (World Spider Catalog 2025). The records from the middle reaches of the Ili River are new for Kazakhstan and represent the easternmost occurrences within the species’ range. The identification was confirmed by D. Logunov.

*Pseudomogrus bactrianus* (Andreeva, 1976) (Fig. 2H and I) was previously known only from Tajikistan (World Spider Catalog 2025). The records from the middle reaches of the Ili River represent the first for Kazakhstan and the easternmost within the species’ range. The identification was confirmed by D. Logunov.

*Pseudomogrus mirabilis* (Logunov & Marusik, 2003) (Fig. 2J, K, Fig. 3G and H) is known from Turkmenistan and Uzbekistan (World Spider Catalog 2025). The record from the Uighur District represents the first for Kazakhstan and the easternmost occurrence within the species’ range. The identification was confirmed by D. Logunov.

*Salticus karakumensis* Logunov & Ponomarev, 2020 (Fig. 2L, M and Fig. 3I-L) has so far been known only from Turkmenistan (World Spider Catalog 2025). The record from the Balkhash Region represents the first for Kazakhstan.

*Xysticus pseudoluctuosus* Marusik & Logunov, 1995 (Fig. 2N, O, Fig. 3M and N) was described from Tajikistan, based on a male (Marusik and Logunov 1995) and later recorded in Turkiye (Demir et al. 2010). The record from the settlement of Talgar is the first for Kazakhstan and the easternmost within the species’ range.

In addition, five more species from this material (*Drassyllus* sp., *Sidydrassus* sp., *Alopecosa* sp., Evippa aff. caucasica Marusik, Guseinov & Koponen, 2003 and Lycosa cf. uzbekistanica Logunov, 2023) are presumably new to science (Table 1).

**Taxonomic and zoogeographic composition of the fauna**

Thus, at least 403 spider species from 158 genera and 31 families are known from the region. The most species-rich family is Salticidae (86 species, 21.3% of the total species richness), followed by Gnaphosidae (53 species, 13.2%) and Lycosidae (46 species, 11.4%). Thomisidae and Araneidae account for 9.7% (39 species) and 7.7% (31 species), respectively, while Linyphiidae, Philodromidae and Theridiidae each contribute about 6% (23–25 species). The share of each of the remaining 23 families does not exceed 2% (1–8 species). The identified faunal structure is generally characteristic of the deserts of Central Asia, in which Salticidae and Gnaphosidae predominate (28% and 15%, respectively), while each of the remaining families accounts for 4–7% of the fauna (*Mikhailov 2013*).

Amongst spiders identified to species level, more than two-thirds (68%, 267 species) have wide Palaearctic or Holarctic distributions. Species with Central Asian and Mediterranean ranges make up 15% (60 species) and 8% (31 species), respectively. Species not found outside Kazakhstan account for 6% (23 species), while the remaining 3% (11 species) include those with a Turanian range as well as species known only from Kazakhstan and Kyrgyzstan or Kazakhstan and Xinjiang (China).

### Discussion

**Comparison of modern, literature and open-source data**

**Figure 4.**
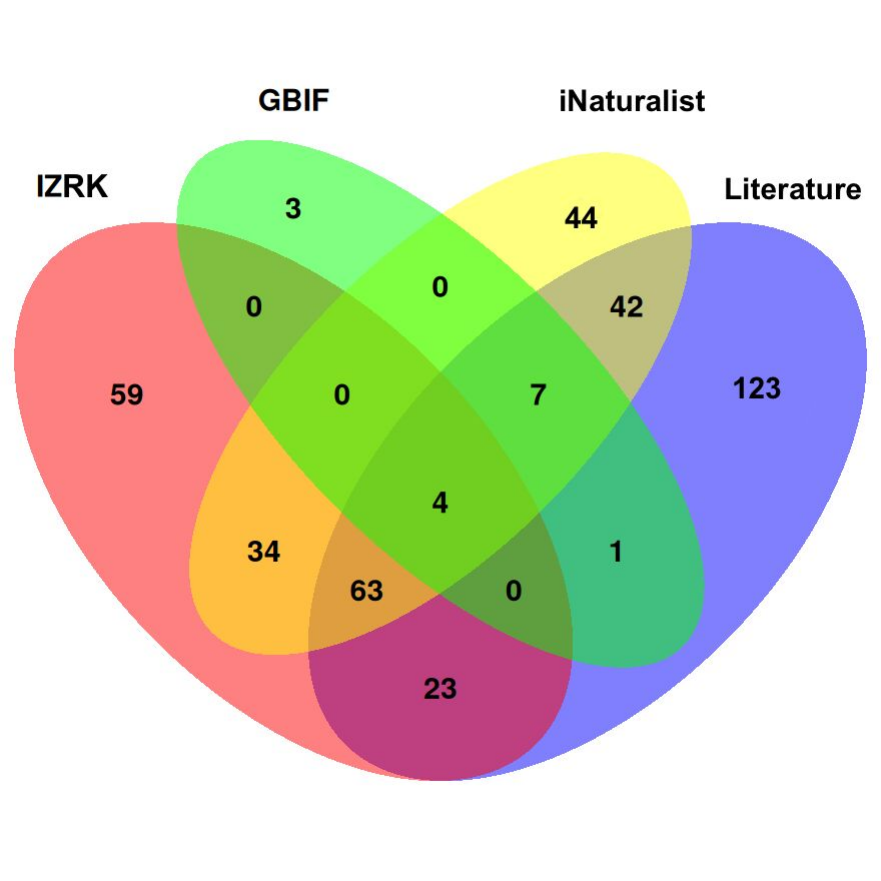
Number of spider species in the considered datasets, including overlap in the species composition.

We present herein the first assessment of spider diversity in the lowland part of the Balkhash-Alakol Basin, based on newly-collected, open-source and previously published data. In addition to differences in the number of species revealed for the region according to different sources (Table 1), these datasets overlap only partially (Fig. 4). More than half of the recorded species (229, 57%) were found exclusively in a single dataset: 123 species only in the literature, 59 in the newly-collected material and 44 and three in iNaturalist and GBIF, respectively.

**Figure 5.**
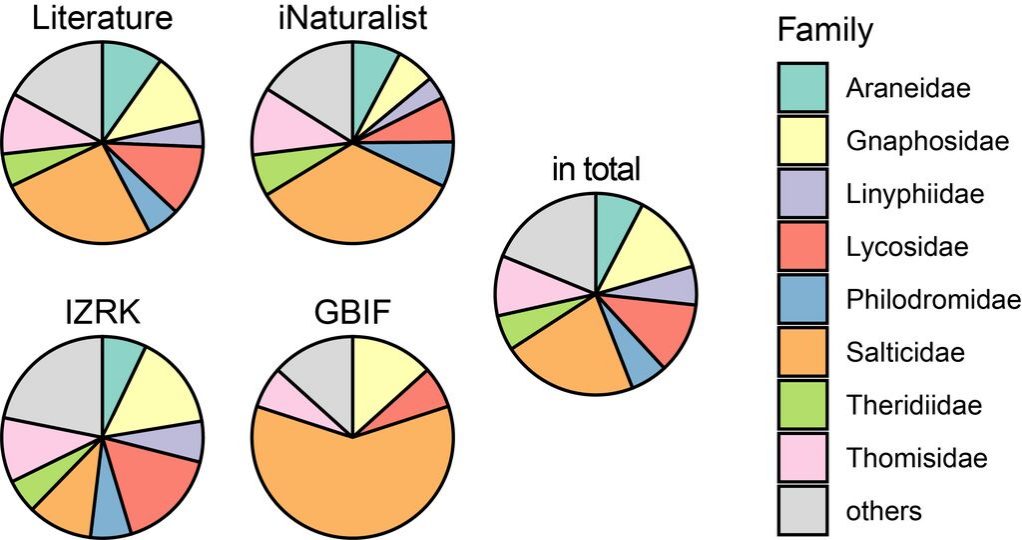
Taxonomical structure (proportion in number of species across families) within datasets considered and the whole fauna.

The compiled checklists reveal an unequal representation of families (qualitative data) (Fig. 5). Thus, in open-source data — provided mainly by nature enthusiasts — the best represented are the most photogenic, conspicuous, colourful and large spiders, such as Salticidae (34% of the total number of species), as well as crab spiders (Thomisidae & Philodromidae) and orb-weavers (Araneidae). Amongst the species collected by professional arachnologists, Lycosidae and Gnaphosidae predominate (16% and 15%, respectively), groups typically collected at night or with pitfall traps and difficult to identify by appearance, along with Thomisidae and Salticidae (10% each). In the checklist compiled from literature data, jumping spiders are also dominant (25%), but Lycosidae (11%) and Gnaphosidae (12%) are well represented as well — unsurprising, given the number and broad temporal coverage of digitised publications.

Thus, each of the compared sources provides only partial faunistic information, as none of them can fully capture the spider diversity of the region. GBIF largely incorporates data from iNaturalist, where most users are not focused on documenting taxonomic diversity and, with rare exceptions, lack the ability to adequately photograph and identify inconspicuous and small spiders (e.g. Gnaphosidae, Lycosidae, Linyphiidae). In contrast, dedicated scientific studies are aimed at obtaining the most comprehensive inventory possible. Unfortunately, the current checklist is still far from complete, as we were unable to survey the entire Balkhash-Alakol Region or cover different seasons of the year. Even the literature-based list, whose family proportions are most similar to those documented for Central Asian deserts (Mikhailov 2013), overlaps with the final checklist by only about two-thirds.

**State of knowledge of the spider fauna of the region**

According to previous estimates, the diversity of spiders inhabiting the arid habitats of south-eastern Kazakhstan, east of the Karatau Ridge (a region approximately twice the size of the Balkhash–Alakol Basin and encompassing it), amounts to at least 262 species (Zyuzin et al. 1995). Our data indicate that the araneofauna of only the lowland and foothill arid part of the Balkhash–Alakol Basin comprises no fewer than 403 species, which represents more than one-third of the spider fauna currently known from Kazakhstan (Mikhailov 2024c).

The revealed fauna is amongst the richest desert faunas in Kazakhstan. For comparison, the araneofauna of the Mangystau Region includes only 195 species (Esyunin et al. 2025), while that of the Kyzylkum Desert comprises at least 188 species (our own data). Nevertheless, considering that our survey of the Balkhash Region did not cover all seasonal aspects of the fauna, as well as the substantial proportion of species new to Kazakhstan or to science (together accounting for 7% of the recorded fauna), we assume that the regional fauna has not yet been fully revealed. It is worth emphasising that the large number of species known exclusively from literature (123) reflects the insufficient level of faunal study in the area rather than changes in species composition over recent decades. In addition, we do not exclude the possibility that some corrections of identifications may have been overlooked during our work and we would greatly appreciate any additions and/or revisions that could help refine the available data.

**Open Data limitations**

It should be noted that we encountered several difficulties when working with data obtained from open sources:

1. Unfavourable angles and/or blurry photographs taken by amateur photographers often make identification difficult (sometimes even at the family level);
2. It is often challenging to separate records (in some cases, a single specimen is uploaded multiple times, contrary to iNaturalist recommendations, which complicates quantitative analysis);
3. Photographs of live spiders often differ considerably from images of specimens preserved in alcohol (both in general habitus and in copulatory organs). For example, brightly-coloured spiders become pale in alcohol and the palps of live versus preserved specimens are difficult to compare due to discrepancies between the palp position in photos and in identification keys, glare on individual sclerites and poor visibility of membranous parts of the palps.

The first two issues can be addressed through content moderation. The last problem may be solved by developing guidelines for standardised photography of live specimens, using a macro lens and diffused lighting to minimise glare. In addition, we would like to draw the attention of professional arachnologists to the fact that photographing specimens both before and after preservation may prove useful when describing new taxa. We also emphasise the high importance of verifying amateur observations, which — given sufficient quality of identifications — can serve as a valuable source of data on fauna, ecology and species distribution.

Despite the challenges mentioned above, it should be noted that the proportion of doubtful records in the data obtained from open sources is relatively low (12%). However, almost all records downloaded from open sources (94%) were uploaded and identified by one of the co-authors of this paper, A. Ozernoy. As a rule, after photographing spiders, he collects them and the actual identification is carried out using collection material (albeit mostly live specimens). For many years, A. Ozernoy has studied the fauna and behaviour of spiders in the Balkhash Region and, unlike most amateurs, has maintained regular contact with specialists. In our view, it is precisely this combination of deep regional knowledge, extensive field experience and scientific collaboration that ensures the high quality and reliability of the presented data.

### Conclusions

Data from open sources can significantly complement both literature and field records, but their use requires caution. The key issues are related to photo quality and angles, duplicate observations and differences between live and preserved specimens. These challenges can be minimised through moderation and standardisation of the photographing process. It is especially important to encourage specialists to verify identifications and naturalists to provide accurate and complete observations — this way, their contributions will become even more valuable and suitable for reliable scientific analysis.

## Supplementary Material

093E15A5-CCA3-573F-9639-623CF18209A4 10.3897/BDJ.14.e181501.suppl1 Supplementary material 1 The articles examined and number of species extracted Data type MS Excel table listing the processed publications Brief description List of processed publications containing information about spiders of the Balkhash-Alakol Basin. File: oo_1413661.xlsx https://binary.pensoft.net/file/1413661 Nekhaeva A.A., Kim L.V., Ishaeva A., Sozontov A.N.

## Figures and Tables

**Table 1.** Checklist of spider species from the lowland and foothill arid parts of the Balkhash-Alakol Basin, compiled from various sources. Species with uncertain identification have been excluded (see comments in the text). Designations: * – species recorded in Kazakhstan for the first time; ** – presumably a species new to science.

**Family**	**Species**	**Literatire**	**iNaturalist**	**GBIF**	**IZ RK**
Agelenidae	*Agelena labyrinthica* (Clerck, 1757)	+	+		
Agelenidae	*Agelena orientalis* C. L. Koch, 1837	+	+		+
Agelenidae	*Allagelena gracilens* (C. L. Koch, 1841)		+		+
Agelenidae	*Benoitia tadzhika* (Andreeva, 1976)		+		
Agelenidae	*Brignoliolus turkestanicus* (Ovtchinnikov, 1999)	+			
Agelenidae	*Eratigena agrestis* (Walckenaer, 1802)		+		+
Agelenidae	*Pireneitega luctuosa* (L. Koch, 1878)	+			
Agelenidae	*Tegenaria domestica* (Clerck, 1757)	+	+		+
Araneidae	*Aculepeira armida* (Audouin, 1826)				+
Araneidae	*Aculepeira carbonaria* (L. Koch, 1869)	+			
Araneidae	*Aculepeira packardi* (Thorell, 1875)	+			
Araneidae	*Agalenatea redii* (Scopoli, 1763)	+	+		
Araneidae	*Araneus alsine* (Walckenaer, 1802)	+			
Araneidae	*Araneus angulatus* Clerck, 1757	+			
Araneidae	*Araneus diadematus* Clerck, 1757	+	+		+
Araneidae	*Araneus grossus* (C. L. Koch, 1844)		+		+
Araneidae	*Araneus marmoreus* Clerck, 1757	+			
Araneidae	*Araneus pallasi* (Thorell, 1875)	+	+		+
Araneidae	*Araneus quadratus* Clerck, 1757	+			
Araneidae	*Araneus strandiellus* Charitonov, 1951	+	+		
Araneidae	*Araneus tartaricus* (Kroneberg, 1875)	+	+		+
Araneidae	*Araniella cucurbitina* (Clerck, 1757)	+			
Araneidae	*Araniella villanii* Zamani, Marusik & Šestáková, 2020		+		
Araneidae	*Argiope bruennichi* (Scopoli, 1772)	+	+		
Araneidae	*Argiope lobata* (Pallas, 1772)	+	+		+
Araneidae	*Cercidia prominens* (Westring, 1851)	+			
Araneidae	*Gibbaranea bituberculata* (Walckenaer, 1802)	+			+
Araneidae	*Gibbaranea ullrichi* (Hahn, 1835)	+	+		
Araneidae	*Hypsosinga pygmaea* (Sundevall, 1831)	+	+		+
Araneidae	*Hypsosinga sanguinea* (C. L. Koch, 1844)	+	+		+
Araneidae	*Larinia chloris* (Audouin, 1826) *				+
Araneidae	*Larinioides cornutus* (Clerck, 1757)	+			
Araneidae	*Larinioides ixobolus* (Thorell, 1873)	+			
Araneidae	*Larinioides patagiatus* (Clerck, 1757)	+			
Araneidae	*Larinioides suspicax* (O. Pickard-Cambridge, 1876)		+		+
Araneidae	*Leviellus stroemi* (Thorell, 1870)	+			
Araneidae	*Mangora acalypha* (Walckenaer, 1802)	+	+		+
Araneidae	*Neoscona adianta* (Walckenaer, 1802)	+	+		+
Araneidae	*Singa hamata* (Clerck, 1757)	+			
Argyronetidae	*Argyroneta aquatica* (Clerck, 1757)	+			
Cheiracanthiidae	*Cheiracanthium pennyi* O. Pickard-Cambridge, 1873				+
Cheiracanthiidae	*Cheiracanthium punctorium* (Villers, 1789)	+			
Cheiracanthiidae	*Cheiracanthium virescens* (Sundevall, 1833) *		+		+
Clubionidae	*Clubiona diversa* O. Pickard-Cambridge, 1862	+			
Clubionidae	*Clubiona germanica* Thorell, 1871	+			
Clubionidae	*Clubiona neglecta* O. Pickard-Cambridge, 1862	+			
Clubionidae	*Clubiona phragmitis* C. L. Koch, 1843	+			
Clubionidae	*Clubiona rybini* Mikhailov, 1992	+			
Clubionidae	*Clubiona subsultans* Thorell, 1875	+			
Clubionidae	*Porrhoclubiona genevensis* (L. Koch, 1866)	+			
Clubionidae	*Porrhoclubiona laudata* (O. Pickard-Cambridge, 1885) *		+		+
Dictynidae	*Archaeodictyna consecuta* (O. Pickard-Cambridge, 1872)				+
Dictynidae	*Brigittea latens* (Fabricius, 1775)	+	+		+
Dictynidae	*Dictyna arundinacea* (Linnaeus, 1758)	+			+
Dictynidae	*Dictynomorpha strandi* Spassky, 1939		+		
Dictynidae	*Shikibutyna wangi* (Song & Zhou, 1986)				+
Dictynidae	*Tolkienus ottoi* (Marusik & Koponen, 2017) *				+
Dysderidae	*Dysdera* sp.		+		+
Dysderidae	*Dysdera tartarica* Kroneberg, 1875				+
Eresidae	*Eresus tristis* Kroneberg, 1875	+			
Eresidae	*Stegodyphus lineatus* (Latreille, 1817)		+		+
Filistatidae	*Pritha* sp.				+
Filistatidae	*Zaitunia logunovi* Zonstein & Marusik, 2016			+	
Filistatidae	*Zaitunia zonsteini* Fomichev & Marusik, 2013			+	
Gnaphosidae	*Aphantaulax trifasciata* (O. Pickard-Cambridge, 1872)		+		+
Gnaphosidae	*Berlandina caspica* Ponomarev, 1979				+
Gnaphosidae	*Berlandina charitonovi* (Ponomarev, 1979)				+
Gnaphosidae	*Berlandina cinerea* (Menge, 1872)	+			
Gnaphosidae	*Berlandina hui* Song, Zhu & Zhang, 2004	+			
Gnaphosidae	*Berlandina ilika* Fomichev & Marusik, 2019	+			+
Gnaphosidae	*Berlandina plumalis* (O. Pickard-Cambridge, 1872)		+		
Gnaphosidae	*Berlandina propinqua* Roewer, 1961	+			
Gnaphosidae	*Berlandina saraevi* Ponomarev, 2008		+		
Gnaphosidae	*Berlandina shnitnikovi* (Spassky, 1934)	+			
Gnaphosidae	*Berlandina spasskyi* Ponomarev, 1979		+		+
Gnaphosidae	*Civizelotes caucasius* (L. Koch, 1866)				+
Gnaphosidae	*Coreodrassus recepsahini* Coşar, Danışman & Marusik, 2024 *				+
Gnaphosidae	*Drassodes chybyndensis* Esyunin & Tuneva, 2002				+
Gnaphosidae	*Drassodes lapidosus* (Walckenaer, 1802)	+			+
Gnaphosidae	*Drassodes longispinus* Marusik & Logunov, 1995 *				+
Gnaphosidae	*Drassodes lutescens* (C. L. Koch, 1839)				+
Gnaphosidae	*Drassyllus lutetianus* (L. Koch, 1866)				+
Gnaphosidae	*Drassyllus praeficus* (L. Koch, 1866)	+	+		+
Gnaphosidae	*Drassyllus* sp. **				+
Gnaphosidae	*Fedotovia uzbekistanica* Charitonov, 1946		+		
Gnaphosidae	*Gnaphosa dolosa* Herman, 1879	+			+
Gnaphosidae	*Gnaphosa fagei* Schenkel, 1963		+		
Gnaphosidae	*Gnaphosa ilika* Ovtsharenko, Platnick & Song, 1992	+			+
Gnaphosidae	*Gnaphosa leporina* (L. Koch, 1866)				+
Gnaphosidae	*Gnaphosa licenti* Schenkel, 1953	+	+		+
Gnaphosidae	*Gnaphosa lucifuga* (Walckenaer, 1802)	+			
Gnaphosidae	*Gnaphosa mongolica* Simon, 1895	+	+		+
Gnaphosidae	*Gnaphosa reikhardi* Ovtsharenko, Platnick & Song, 1992	+			
Gnaphosidae	*Gnaphosa taurica* Thorell, 1875	+			
Gnaphosidae	*Haplodrassus ovtchinnikovi* Ponomarev, 2008				+
Gnaphosidae	*Haplodrassus rugosus* Tuneva, 2004	+			
Gnaphosidae	*Haplodrassus signifer* (C. L. Koch, 1839)		+		
Gnaphosidae	*Heser malefactor* Tuneva, 2004	+			
Gnaphosidae	*Marinarozelotes fuscipes* (L. Koch, 1866)	+			
Gnaphosidae	*Marinarozelotes lyonneti* (Audouin, 1826)				+
Gnaphosidae	*Micaria formicaria* (Sundevall, 1831)	+			
Gnaphosidae	*Micaria fulgens* (Walckenaer, 1802)	+			
Gnaphosidae	*Micaria lenzi* Bösenberg, 1899	+			
Gnaphosidae	*Micaria pulicaria* (Sundevall, 1831)	+			
Gnaphosidae	*Micaria rossica* Thorell, 1875	+	+		+
Gnaphosidae	*Nomisia aussereri* (L. Koch, 1872)	+	+		+
Gnaphosidae	*Sidydrassus shumakovi* (Spassky, 1934)	+		+	
Gnaphosidae	*Sidydrassus* sp. **				+
Gnaphosidae	*Sidydrassus tianschanicus* (Hu & Wu, 1989)	+	+	+	+
Gnaphosidae	*Sosticus loricatus* (L. Koch, 1866)	+			
Gnaphosidae	*Synaphosus palearcticus* Ovtsharenko, Levy & Platnick, 1994	+			
Gnaphosidae	*Synaphosus taukum* Ovtsharenko, Levy & Platnick, 1994	+			
Gnaphosidae	*Synaphosus turanicus* Ovtsharenko, Levy & Platnick, 1994	+			
Gnaphosidae	*Talanites involutus* (O. Pickard-Cambridge, 1885)	+			
Gnaphosidae	*Urozelotes rusticus* (L. Koch, 1872) *				+
Gnaphosidae	*Zelotes atrocaeruleus* (Simon, 1878)				+
Gnaphosidae	*Zelotes longipes* (L. Koch, 1866)	+			+
Hersiliidae	Hersiliola cf. korbi Fomichev, 2025 *				+
Hersiliidae	*Hersiliola xinjiangensis* (Liang & Wang, 1989) *		+		+
Linyphiidae	*Agyneta fuscipalpus* (C. L. Koch, 1836)				+
Linyphiidae	*Agyneta rurestris* (C. L. Koch, 1836)				+
Linyphiidae	*Agyneta simplicitarsis* (Simon, 1884)				+
Linyphiidae	*Agyneta* sp.				+
Linyphiidae	Bolephthyphantes cf. indexoides (Tanasevitch, 1989)		+		
Linyphiidae	*Caviphantes dobrogicus* (Dumitrescu & Miller, 1962) *				+
Linyphiidae	*Ceratinella brevis* (Wider, 1834)				+
Linyphiidae	*Diplostyla concolor* (Wider, 1834)				+
Linyphiidae	*Entelecara acuminata* (Wider, 1834)	+	+		
Linyphiidae	Entelecara cf. erythropus (Westring, 1851)		+		
Linyphiidae	*Erigone atra* Blackwall, 1833	+			
Linyphiidae	*Erigone dentipalpis* (Wider, 1834)	+	+		+
Linyphiidae	*Hylyphantes* sp.	+			
Linyphiidae	*Ipa pepticus* (Tanasevitch, 1988)	+			
Linyphiidae	*Lepthyphantes leprosus* (Ohlert, 1865)	+			
Linyphiidae	*Megalepthyphantes kronebergi* (Tanasevitch, 1989)		+		
Linyphiidae	*Megalepthyphantes nebulosus* (Sundevall, 1830)		+		
Linyphiidae	*Microlinyphia pusilla* (Sundevall, 1830)	+			+
Linyphiidae	*Neriene clathrata* (Sundevall, 1830)	+			
Linyphiidae	*Neriene montana* (Clerck, 1757)	+			
Linyphiidae	*Oedothorax apicatus* (Blackwall, 1850)	+			
Linyphiidae	*Pityohyphantes phrygianus* (C. L. Koch, 1836)	+			
Linyphiidae	*Stemonyphantes lineatus* (Linnaeus, 1758)		+		+
Linyphiidae	*Tenuiphantes tenuis* (Blackwall, 1852)				+
Linyphiidae	*Vagiphantes vaginatus* (Tanasevitch, 1983)				+
Liocranidae	*Agroeca cuprea* Menge, 1873	+			
Liocranidae	*Agroeca lusatica* (L. Koch, 1875)	+			
Lycosidae	*Alopecosa albofasciata* (Brullé, 1832)	+			
Lycosidae	*Alopecosa cuneata* (Clerck, 1757)	+			+
Lycosidae	*Alopecosa cursor* (Hahn, 1831)	+			+
Lycosidae	*Alopecosa marikovskyi* Logunov, 2013	+	+		+
Lycosidae	*Alopecosa pulverulenta* (Clerck, 1757)	+			
Lycosidae	*Alopecosa schmidti* (Hahn, 1835)				+
Lycosidae	*Alopecosa* sp. **		+		+
Lycosidae	*Alopecosa taeniopus* (Kulczyński, 1895)	+	+		+
Lycosidae	*Arctosa cinerea* (Fabricius, 1777)				+
Lycosidae	*Arctosa leopardus* (Sundevall, 1833)	+	+		
Lycosidae	*Arctosa stigmosa* (Thorell, 1875)				+
Lycosidae	*Bogdocosa kronebergi* (Andreeva, 1976)		+		+
Lycosidae	*Evippa beschkentica* Andreeva, 1976 *				+
Lycosidae	Evippa aff. caucasica Marusik, Guseinov & Koponen, 2003		+		+
Lycosidae	*Evippa onager* Simon, 1895 sensu Šternbergs 1979 *				+
Lycosidae	*Evippa sjostedti* Schenkel, 1936		+		+
Lycosidae	*Evippa turkmenica* Sternbergs, 1979				+
Lycosidae	*Halocosa cereipes* (L. Koch, 1878)				+
Lycosidae	*Karakumosa alticeps* (Kroneberg, 1875)	+	+	+	+
Lycosidae	*Karakumosa xinjiang* Wang, Yang & Zhang, 2023 *				+
Lycosidae	*Lycosa praegrandis* C. L. Koch, 1836	+	+		+
Lycosidae	*Lycosa singoriensis* (Laxmann, 1770)	+	+		+
Lycosidae	Lycosa cf. uzbekistanica Logunov, 2023 **				+
Lycosidae	*Pardosa agrestis* (Westring, 1861)	+			+
Lycosidae	*Pardosa agricola* (Thorell, 1856)	+			+
Lycosidae	*Pardosa amentata* (Clerck, 1757)	+			
Lycosidae	*Pardosa atrata* (Thorell, 1873)	+			
Lycosidae	*Pardosa fortunata* (O. Pickard-Cambridge, 1885)	+			
Lycosidae	*Pardosa gromovi* Ballarin, Marusik, Omelko & Koponen, 2012	+			+
Lycosidae	*Pardosa italica* Tongiorgi, 1966	+			
Lycosidae	*Pardosa jaikensis* Ponomarev, 2007		+		+
Lycosidae	*Pardosa jergeniensis* Ponomarev, 1979	+			
Lycosidae	*Pardosa luctinosa* Simon, 1876	+			
Lycosidae	*Pardosa mikhailovi* Ballarin, Marusik, Omelko & Koponen, 2012	+			+
Lycosidae	*Pardosa nebulosa* (Thorell, 1872)	+	+		+
Lycosidae	*Pardosa paludicola* (Clerck, 1757)	+	+		
Lycosidae	*Pardosa palustris* (Linnaeus, 1758)	+			
Lycosidae	*Pardosa pullata* (Clerck, 1757)	+			
Lycosidae	*Pardosa riparia* (C. L. Koch, 1833)	+			
Lycosidae	*Pardosa turkestanica* (Roewer, 1951)	+			
Lycosidae	*Pardosa zonsteini* Ballarin, Marusik, Omelko & Koponen, 2012				+
Lycosidae	*Pirata* sp.	+			
Lycosidae	*Piratula hygrophila* (Thorell, 1872)	+			
Lycosidae	*Trochosa robusta* (Simon, 1876)				+
Lycosidae	*Trochosa ruricola* (De Geer, 1778)	+			+
Lycosidae	*Xerolycosa miniata* (C. L. Koch, 1834)	+	+		+
Mimetidae	*Ero aphana* (Walckenaer, 1802)		+		
Mimetidae	*Mimetus laevigatus* (Keyserling, 1863)		+		+
Miturgidae	*Zora pardalis* Simon, 1878	+			
Miturgidae	*Zora spinimana* (Sundevall, 1833)	+			
Oecobiidae	*Oecobius nadiae* (Spassky, 1936)		+		+
Oecobiidae	*Turanobius ferdowsii* (Mirshamsi, Zamani & Marusik, 2017)				+
Oecobiidae	*Turanobius leptonychus* Zamani, Marusik & Fomichev, 2024 *				+
Oxyopidae	*Oxyopes globifer* Simon, 1876		+		+
Oxyopidae	*Oxyopes heterophthalmus* (Latreille, 1804)	+			
Oxyopidae	*Oxyopes lineatus* Latreille, 1806	+	+		+
Oxyopidae	*Oxyopes nenilini* Esyunin & Tuneva, 2009		+		
Oxyopidae	*Oxyopes takobius* Andreeva & Tystshenko, 1969		+		
Philodromidae	*Philodromus aureolus* (Clerck, 1757)	+			
Philodromidae	*Philodromus buxi* Simon, 1884				+
Philodromidae	*Philodromus cespitum* (Walckenaer, 1802)		+		+
Philodromidae	Philodromus cf. longipalpis Simon, 1870 *		+		
Philodromidae	*Philodromus poecilus* (Thorell, 1872)	+	+		
Philodromidae	*Rhysodromus ablegminus* (Szita & Logunov, 2008)	+	+		
Philodromidae	*Rhysodromus alascensis* (Keyserling, 1884)	+			
Philodromidae	*Rhysodromus fallax* (Sundevall, 1833)	+	+		
Philodromidae	*Rhysodromus histrio* (Latreille, 1819)	+			
Philodromidae	*Rhysodromus pictus* (Kroneberg, 1875)	+	+		+
Philodromidae	*Rhysodromus timidus* (Szita & Logunov, 2008)	+			+
Philodromidae	*Rhysodromus triangulatus* (Urita & Song, 1987)	+			
Philodromidae	*Rhysodromus xerophilus* (Szita & Logunov, 2008)	+			
Philodromidae	*Rhysodromus xinjiangensis* (Tang & Song, 1987)	+			
Philodromidae	*Thanatus formicinus* (Clerck, 1757)		+		+
Philodromidae	*Thanatus imbecillus* L. Koch, 1878		+		
Philodromidae	*Thanatus jaikensis* Ponomarev, 2007				+
Philodromidae	*Thanatus kitabensis* Charitonov, 1946	+	+		+
Philodromidae	*Thanatus mikhailovi* Logunov, 1996		+		
Philodromidae	*Thanatus oblongiusculus* (Lucas, 1846)	+	+		+
Philodromidae	*Thanatus pictus* L. Koch, 1881		+		+
Philodromidae	*Thanatus sabulosus* (Menge, 1875)				+
Philodromidae	*Thanatus vulgaris* Simon, 1870		+		+
Philodromidae	*Tibellus oblongus* (Walckenaer, 1802)	+	+		+
Pholcidae	*Pholcus arkit* Huber, 2011	+			
Pholcidae	*Pholcus manueli* Gertsch, 1937	+	+		+
Pholcidae	*Pholcus opilionoides* (Schrank, 1781)	+			
Pholcidae	*Pholcus ponticus* Thorell, 1875	+	+		+
Pholcidae	*Pholcus sogdianae* Brignoli, 1978	+			+
Pisauridae	*Dolomedes fimbriatus* (Clerck, 1757)	+			
Pisauridae	*Pisaura mirabilis* (Clerck, 1757)	+	+		+
Salticidae	*Aelurillus andreevae* Nenilin, 1984 *		+		
Salticidae	*Aelurillus ater* (Kroneberg, 1875)			+	
Salticidae	*Aelurillus concolor* Kulczyński, 1901		+		
Salticidae	*Aelurillus dubatolovi* Azarkina, 2003		+		
Salticidae	*Aelurillus m-nigrum* Kulczyński, 1891	+	+		
Salticidae	Aelurillus cf. nenilini Azarkina, 2002		+		
Salticidae	*Aelurillus v-insignitus* (Clerck, 1757)	+	+		+
Salticidae	*Attulus avocator* (O. Pickard-Cambridge, 1885)	+	+		+
Salticidae	*Attulus fasciger* (Simon, 1880) *		+		+
Salticidae	*Attulus inexpectus* (Logunov & Kronestedt, 1997)	+	+		+
Salticidae	*Attulus inopinabilis* (Logunov, 1992)	+			
Salticidae	*Attulus kazakhstanicus* (Logunov, 1992)	+			
Salticidae	*Attulus mirandus* (Logunov, 1993)	+	+		
Salticidae	*Attulus nenilini* (Logunov & Wesołowska, 1993)	+	+		
Salticidae	*Attulus terebratus* (Clerck, 1757)	+			
Salticidae	*Attulus zimmermanni* (Simon, 1877)		+		
Salticidae	*Ballus chalybeius* (Walckenaer, 1802)		+		+
Salticidae	*Chalcoscirtus brevicymbialis* Wunderlich, 1980	+			
Salticidae	*Chalcoscirtus infimus* (Simon, 1868)	+	+		
Salticidae	*Chalcoscirtus karakurt* Marusik, 1991	+	+		
Salticidae	*Chalcoscirtus nigritus* (Thorell, 1875)	+	+		+
Salticidae	*Chalcoscirtus paraansobicus* Marusik, 1990	+			
Salticidae	*Chalcoscirtus parvulus* Marusik, 1991	+			
Salticidae	*Chalcoscirtus platnicki* Marusik, 1995	+			
Salticidae	*Chalcoscirtus tanasevichi* Marusik, 1991	+			
Salticidae	*Euophrys frontalis* (Walckenaer, 1802)	+	+		
Salticidae	*Euophrys uralensis* Logunov, Cutler & Marusik, 1993	+	+		
Salticidae	*Evarcha arcuata* (Clerck, 1757)	+	+		+
Salticidae	*Heliophanus auratus* C. L. Koch, 1835	+	+		+
Salticidae	*Heliophanus chovdensis* Prószyński, 1982	+	+		+
Salticidae	*Heliophanus curvidens* (O. Pickard-Cambridge, 1872)	+	+		+
Salticidae	*Heliophanus flavipes* (Hahn, 1832)	+			
Salticidae	*Heliophanus forcipifer* Kulczyński, 1895	+	+		
Salticidae	*Heliophanus patagiatus* Thorell, 1875	+	+		
Salticidae	*Heliophanus potanini* Schenkel, 1963	+	+		+
Salticidae	*Heliophanus wesolowskae* Rakov & Logunov, 1997		+		
Salticidae	*Marpissa pomatia* (Walckenaer, 1802)	+	+		
Salticidae	*Marusyllus aralicus* (Logunov & Marusik, 2003)		+		
Salticidae	*Marusyllus coreanus* (Prószyński, 1968)	+	+		
Salticidae	*Marusyllus uzbekistanicus* (Logunov & Marusik, 2003)		+		
Salticidae	*Mogrus antoninus* Andreeva, 1976	+	+		
Salticidae	*Mogrus larisae* Logunov, 1995	+	+		
Salticidae	*Mogrus neglectus* (Simon, 1868)	+			
Salticidae	*Mogrus valerii* Kononenko, 1981 *		+		
Salticidae	*Pellenes allegrii* Caporiacco, 1935	+	+		
Salticidae	*Pellenes amazonka* Logunov, Marusik & Rakov, 1999	+			
Salticidae	*Pellenes dilutus* Logunov, 1995		+		
Salticidae	*Pellenes epularis* (O. Pickard-Cambridge, 1872)	+	+		
Salticidae	*Pellenes geniculatus* (Simon, 1868)	+	+		
Salticidae	*Pellenes seriatus* (Thorell, 1875)	+	+		
Salticidae	*Philaeus chrysops* (Poda, 1761)	+	+		+
Salticidae	*Phlegra andreevae* Logunov, 1996	+	+		
Salticidae	*Phlegra cinereofasciata* (Simon, 1868)	+			
Salticidae	*Phlegra fasciata* (Hahn, 1826)	+	+		+
Salticidae	*Phlegra obscurimagna* Azarkina, 2004	+			
Salticidae	*Phlegra profuga* Logunov, 1996	+			
Salticidae	*Pseudeuophrys obsoleta* (Simon, 1868)	+	+		+
Salticidae	*Pseudicius courtauldi* Bristowe, 1935	+	+		+
Salticidae	*Pseudicius encarpatus* (Walckenaer, 1802)	+	+		
Salticidae	*Pseudomogrus albocinctus* (Kroneberg, 1875)	+	+		
Salticidae	*Pseudomogrus bactrianus* (Andreeva, 1976) *		+		
Salticidae	*Pseudomogrus bakanas* (Logunov & Marusik, 2003)	+	+		
Salticidae	*Pseudomogrus dalaensis* (Logunov & Marusik, 2003)	+	+	+	+
Salticidae	*Pseudomogrus guseinovi* (Logunov & Marusik, 2003)		+		+
Salticidae	*Pseudomogrus mirabilis* (Logunov & Marusik, 2003) *		+		
Salticidae	*Pseudomogrus pseudovalidus* (Logunov & Marusik, 2003)	+	+	+	
Salticidae	*Pseudomogrus validus* (Simon, 1889)	+	+		
Salticidae	*Pseudomogrus vittatus* (Thorell, 1875)		+		
Salticidae	*Pseudomogrus zhilgaensis* (Logunov & Marusik, 2003)	+	+	+	
Salticidae	*Rafalus variegatus* (Kroneberg, 1875)		+		
Salticidae	*Rudakius afghanicus* (Andreeva, Hęciak & Prószyński, 1984)	+			
Salticidae	*Rudakius cinctus* (O. Pickard-Cambridge, 1885)	+	+		+
Salticidae	*Salticus dzhungaricus* Logunov, 1992	+	+		
Salticidae	*Salticus karakumensis* Logunov & Ponomarev, 2020 *		+		+
Salticidae	*Salticus proszynskii* Logunov, 1992	+			
Salticidae	*Salticus tricinctus* (C. L. Koch, 1846)	+	+	+	
Salticidae	*Synageles charitonovi* Andreeva, 1976		+		
Salticidae	*Synageles subcingulatus* (Simon, 1878)	+	+		
Salticidae	*Talavera aperta* (Miller, 1971)	+			
Salticidae	*Talavera krocha* Logunov & Kronestedt, 2003	+			
Salticidae	*Talavera petrensis* (C. L. Koch, 1837)	+	+		
Salticidae	*Talavera thorelli* (Kulczyński, 1891)	+			
Salticidae	*Yllenus dunini* Logunov & Marusik, 2003	+	+	+	
Salticidae	*Yllenus turkestanicus* Logunov & Marusik, 2003	+	+	+	
Salticidae	*Yllenus uiguricus* Logunov & Marusik, 2003	+	+	+	
Salticidae	*Yllenus zyuzini* Logunov & Marusik, 2003	+	+	+	
Scytodidae	*Scytodes univittata* Simon, 1882 *				+
Segestriidae	*Segestria* sp.	+			
Sparassidae	*Cebrennus kazakhstanicus* Fomichev & Marusik, 2022	+	+		+
Sparassidae	*Micrommata virescens* (Clerck, 1757)	+	+		
Sparassidae	*Olios sericeus* (Kroneberg, 1875)	+	+		+
Tetragnathidae	*Metleucauge dentipalpis* (Kroneberg, 1875)	+			
Tetragnathidae	*Pachygnatha clercki* Sundevall, 1823	+			
Tetragnathidae	*Pachygnatha degeeri* Sundevall, 1830	+			+
Tetragnathidae	*Tetragnatha extensa* (Linnaeus, 1758)	+	+		
Tetragnathidae	*Tetragnatha montana* Simon, 1874	+			+
Tetragnathidae	*Tetragnatha obtusa* C. L. Koch, 1837	+			
Tetragnathidae	*Tetragnatha pinicola* L. Koch, 1870	+	+		+
Theridiidae	*Asagena phalerata* (Panzer, 1801)	+	+		+
Theridiidae	*Asagena semideserta* (Ponomarev, 2005)	+			
Theridiidae	Enoplognatha cf. latimana Hippa & Oksala, 1982		+		
Theridiidae	*Enoplognatha ovata* (Clerck, 1757)	+			
Theridiidae	*Enoplognatha submargarita* Yaginuma & Zhu, 1992		+		+
Theridiidae	*Euryopis laeta* (Westring, 1861)	+			
Theridiidae	*Euryopis saukea* Levi, 1951		+		
Theridiidae	*Latrodectus tredecimguttatus* (Rossi, 1790)	+	+		+
Theridiidae	Neottiura cf. bimaculata (Linnaeus, 1767)		+		
Theridiidae	*Paidiscura dromedaria* (Simon, 1880)	+			
Theridiidae	*Parasteatoda tabulata* (Levi, 1980)	+			
Theridiidae	*Parasteatoda tepidariorum* (C. L. Koch, 1841)		+		+
Theridiidae	*Phylloneta impressa* (L. Koch, 1881)	+	+		+
Theridiidae	*Phylloneta sisyphia* (Clerck, 1757)				+
Theridiidae	*Steatoda albomaculata* (De Geer, 1778)	+	+		+
Theridiidae	*Steatoda bipunctata* (Linnaeus, 1758)	+			
Theridiidae	*Steatoda castanea* (Clerck, 1757)	+	+		+
Theridiidae	*Steatoda grossa* (C. L. Koch, 1838)		+		
Theridiidae	*Steatoda paykulliana* (Walckenaer, 1806)	+	+		+
Theridiidae	*Steatoda* sp.				+
Theridiidae	*Theridion melanurum* Hahn, 1831	+			
Theridiidae	Theridion cf. mystaceum L. Koch, 1870		+		
Theridiidae	*Theridion varians* Hahn, 1833	+			
Thomisidae	*Bassaniodes graecus* (C. L. Koch, 1837)	+			
Thomisidae	*Bassaniodes loeffleri* (Roewer, 1955)	+			+
Thomisidae	*Bassaniodes robustus* (Hahn, 1832)	+			+
Thomisidae	*Bassaniodes tristrami* (O. Pickard-Cambridge, 1872)	+	+	+	+
Thomisidae	Bassaniodes cf. turlan (Marusik & Logunov, 1990)		+		
Thomisidae	*Diaea dorsata* (Fabricius, 1777)	+			
Thomisidae	*Diaea suspiciosa* O. Pickard-Cambridge, 1885		+		+
Thomisidae	*Ebrechtella tricuspidata* (Fabricius, 1775)	+	+		+
Thomisidae	*Heriaeus capillatus* Utochkin, 1985	+			
Thomisidae	*Heriaeus hirtus* (Latreille, 1819)	+			
Thomisidae	*Heriaeus mellotteei* Simon, 1886				+
Thomisidae	*Misumena vatia* (Clerck, 1757)	+	+		
Thomisidae	*Misumenops armatus* Spassky, 1952		+		
Thomisidae	*Ozyptila inaequalis* (Kulczyński, 1901)	+			
Thomisidae	*Ozyptila lugubris* (Kroneberg, 1875)	+	+		+
Thomisidae	*Ozyptila praticola* (C. L. Koch, 1837)	+	+		+
Thomisidae	*Ozyptila scabricula* (Westring, 1851)	+	+		+
Thomisidae	Ozyptila cf. tuberosa (Thorell, 1875)		+		
Thomisidae	*Psammitis marmorata* (Thorell, 1875)		+		+
Thomisidae	*Psammitis minor* (Charitonov, 1946)	+	+		
Thomisidae	*Psammitis ninnii* (Thorell, 1872)	+			
Thomisidae	*Psammitis tyshchenkoi* (Marusik & Logunov, 1995)				+
Thomisidae	*Spiracme striatipes* (L. Koch, 1870)		+		+
Thomisidae	*Synema plorator* (O. Pickard-Cambridge, 1872)	+			
Thomisidae	*Synema utotchkini* Marusik & Logunov, 1995	+	+		
Thomisidae	*Thomisus onustus* Walckenaer, 1805	+	+		+
Thomisidae	*Xysticus bakanas* Marusik & Logunov, 1990	+			+
Thomisidae	*Xysticus bifasciatus* C. L. Koch, 1837	+			
Thomisidae	*Xysticus ephippiatus* Simon, 1880		+		
Thomisidae	Xysticus cf. kuzgi Marusik & Logunov, 1990		+		
Thomisidae	*Xysticus lapidarius* Utochkin, 1968	+	+		+
Thomisidae	*Xysticus luctuosus* (Blackwall, 1836)	+			
Thomisidae	*Xysticus mongolicus* Schenkel, 1963	+	+		+
Thomisidae	*Xysticus pseudocristatus* Azarkina & Logunov, 2001	+	+		+
Thomisidae	*Xysticus pseudoluctuosus* Marusik & Logunov, 1995 *		+		
Thomisidae	*Xysticus* sp.				+
Thomisidae	*Xysticus taukumkurt* Marusik & Logunov, 1990	+			
Thomisidae	*Xysticus urgumchak* Marusik & Logunov, 1990	+			
Thomisidae	*Xysticus xerodermus* Strand, 1913 *				+
Titanoecidae	*Nurscia albosignata* Simon, 1874	+	+		+
Titanoecidae	*Titanoeca quadriguttata* (Hahn, 1833)				+
Titanoecidae	*Titanoeca turkmenia* Wunderlich, 1995				+
Uloboridae	*Uloborus walckenaerius* Latreille, 1806	+	+		
Zodariidae	*Zodariellum asiaticum* (Tystshenko, 1970)	+			+
Zodariidae	*Zodariellum martinae* Shafaie & Pekár, 2025	+			
Zodariidae	*Zodariellum nenilini* (Eskov, 1995)		+		+
Zodariidae	*Zodariellum volgouralense* Ponomarev, 2007				+
	**Total**	**263**	**194**	**15**	**183**
